# Genetic Relationships Among Portuguese Cultivated and Wild *Vitis vinifera* L. Germplasm

**DOI:** 10.3389/fpls.2020.00127

**Published:** 2020-03-05

**Authors:** Jorge Cunha, Javier Ibáñez, Margarida Teixeira-Santos, João Brazão, Pedro Fevereiro, José M. Martínez-Zapater, José E. Eiras‐Dias

**Affiliations:** ^1^Instituto Nacional de Investigação Agrária e Veterinária, Dois Portos, Portugal; ^2^Green-it Unit, Instituto de Tecnologia Química e Biológica, Universidade Nova de Lisboa, Oeiras, Portugal; ^3^Departamento de Viticultura, Instituto de Ciencias de la Vid y del Vino, (Consejo Superior de Investigaciones Científicas, Universidad de La Rioja, Gobierno de La Rioja), Logroño, Spain; ^4^Departamento de Biologia Vegetal, Faculdade de Ciências, Universidade de Lisboa, Lisboa, Portugal

**Keywords:** grapevine varieties, genetic relationships, Iberia, introgression, pedigrees, *sylvestris*, wild

## Abstract

The domesticated grapevine spread along the Mediterranean basin from the primary Near East domestication area, where the greatest genetic diversity is found in its ancestor, the wild vine populations. Portuguese wild populations are on the southwestern fringe of the distribution of the *Vitis vinifera* L. ssp*. sylvestris (C.C. Gmel.) Hegi* in Europe. During the last Glacial Period they became isolated from the previous continuum that had been the territory of wild vine populations. Archaeological remains of domesticated *vinifera* grapevines in Portugal date back from 795 Before Common Era (BCE) in the lower Tagus river basin. In this work, 258 Portuguese *vinifera* varieties and *sylvestris* plants were characterized using 261 single nucleotide polymorphism (SNP) markers. The study of the genetic diversity of this local germplasm, its population structure and kinship, all framed in their historical and geographical backgrounds, revealed a complex network of first-degree relationships, where only Iberian varieties are involved. Some Iberian genotypes, like Alfrocheiro (Bruñal, in Spain), Sarigo (Cayetana Blanca), Mourisco Branco (Hebén), Amaral (Caiño Bravo), and Marufo (Moravia Dulce) are ancestors of a considerable fraction of all the autochthonous analyzed varieties. A part of the diversity developed was mostly local in some cases as shown by the closeness of several varieties (Vinhos Verdes) to the wild cluster in different analyses. Besides, several evidences of introgression of domesticated germplasm into wild vines was found, substantiating the high risk of genetic contamination of the *sylvestris* subspecies. All these findings together to the known matching between the wild maternal lineage of the Iberian Peninsula and an important number of Portuguese grapevine varieties (chlorotype A), point out that some of these varieties derive, directly or indirectly, from originally local wild populations, supporting the possible occurrence of secondary events of local domestication, or, at least, of an introgression process of wild into cultivated grapevines.

## Introduction

The domesticated grapevine (*Vitis vinifera* L. ssp. *vinifera*) is the most cultivated fruit crop of the *Vitis* genus, which contains about 60 inter-fertile wild species (This et al., 2006; Emanuelli et al., 2013). *V. vinifera* ssp. *sylvestris* is the only wild *Vitis* taxon native to Europe and the Near East, and it is believed to be the wild progenitor of almost 10,000 domesticated grapevine varieties today (This et al., 2006; Emanuelli et al., 2013). Genetic hybridizations among wild, cultivated and feral types make it difficult to untangle the history of current grapevine varieties (Zohary and Hopf, 1994).

Domestication results from selecting and propagating plants with desirable traits along generations. Given the dioecious behavior of the wild species, and its high heterozygosity, vegetative propagation is believed to have been adopted early in grapevine domestication (Zohary and Hopf, 1994), as the easiest way to maintain desirable traits. The domestication of grapevine generated notable morphological changes, including perfect hermaphrodite flowers, higher sugar content, and larger berry size together with others like altered seed morphology resulting from automatic selection (Olmo, 1995; This et al., 2006; Miller, 2008). For instance, since self-pollinating plants have improved fruit set and are more productive (Miller, 2008), hermaphrodite variant vines were selected compared to female plants. Domesticated grapevines were disseminated along with the spreading of wine culture from their primary domestication sites in the Near East in a process that lasted over 5000 years (McGovern et al., 2017) but likely a reduced number of sexual generations, given their vegetative multiplication (Arroyo-García et al., 2006). Along this time, introgression from local populations or even secondary domestication events could have taken place as suggested by Arroyo-García et al. (2006); Imazio et al. (2006); or Myles et al. (2011).

Relictic wild vine populations can still be found in Portugal in what can be considered as the southwestern fringe of the distribution of the *V. vinifera* ssp. *sylvestris* in Europe. During the late Ice Age, Iberian *Vitis* populations became isolated from the previous continuum that had been the territory of Mediterranean populations. Palynological evidences from Southern Portugal show the presence of pollen from *V. vinifera* in several river basins and lagoons in the Holocene (Leeuwaarden and Janssen, 1985; van der Knaap and van Leeuwen, 1995; Fletcher et al., 2007; Vis and Kasse, 2009; Schneider et al., 2016). Evidences of strong anthropogenic effects on the environment registered on the vegetation of this region after 2090 Before Common Era (BCE) suggest the possibility of domestication/cultivation of grapevine (Fletcher et al., 2007).

The oldest archeological remains of grapevine seeds in Portugal date back from the Chalcolithic (*circa* 3350–2250 BCE) and its presence continues until the Roman time, covering the Bronze and Iron Age and the Phoenician settlements (Ramil Rego and Aira Rodríguez, 1993; Aira Rodríguez and Ramil Rego, 1995; Arruda, 2008). Stummer (1911) introduced an index (width to length ratio) for the distinction between wild and cultivated grapevine seeds. Indexes between 0.76 and 0.83 indicate wild plants, while indices between 0.44 and 0.53 correspond to cultivated plants; values between 0.54 and 0.75 correspond to margins of overlap of the two subspecies. This index has been the main criterion used in the identification of archeological seeds (Renfrew, 1973).

In the Iberian Peninsula the production of wine is well establish since the 7th century BCE (Buxó, 2008). In Portugal, the production and use of grapes in a Phoenician urban context is established by the presence of grapevine seeds in Almada (near Lisbon), an archeological site from the 7th century BCE (Barros, 1998). Ceramic remains containing wine were found in a Phoenician archeological context in Santarem, Almada, and Lisbon (Barros, 1998; Sousa and Guerra, 2018). The 2^nd^ century BCE the Greek author Polybius in his *The Histories* (book 34 chapter 8) refers to the low price of wine in Lusitania (“A metreta of wine costs a drachma”—*circa* 40 liters cost the day payment of a soldier). In the 1^st^ century BCE the also Greek author Strabo in his Geography, book 3 chapter 3, refers to vineyards in the lower Tagus valley and in chapter 2 to wine exports from Tudertania. Roman remains (1^st^ century BCE to 5th century CE) are ubiquitous, including grape seeds remains in archeological sites, oenological equipment and amphorae used to storage and transport of wine (Fabião, 1998; Tereso et al., 2011; Savo et al., 2016). Viticulture was not abandoned after the defeat of the Visigoths kingdom by Tariq (711 CE), and later in the Middle Ages, viticulture spread throughout the country likely related to the expansion of Christian kingdoms and also accompanied by the establishment of several monastic orders (Benedictines and Cistercians) with strong viticulture traditions (Fernandes, 1532; Martinez Tomé, 1991).

Ampelography (Aµπϵλος, “vine” and γραφος, “writing”) is the science of identifying, naming, and classifying grape varieties, mainly through its phenotypic characterization, and ampelographic data is the base of the description and identification of grapevines (Galet, 1979). Well-established ampelographic descriptors from the International Organization of Vine and Wine (OIV, 1983; OIV, 2009) identified numerous synonym and homonym varieties. However, the subjectivity of individual observations, as well as the variability generated by plant growing conditions, sanitary status and growing season, prevented unambiguous identifications. Single nucleotide polymorphisms (SNP) are one of the most powerful DNA markers recently developed. They have been adapted to a large number of applications in the study of grapevine, including genetic identification (Cabezas et al., 2011; Cunha et al., 2016), diversity studies, genetic structure, and domestication history of grape (Myles et al., 2011), or pedigree and phylogeny studies (Ibáñez et al., 2012; Zinelabidine et al., 2012). SNPs are alternative or complementary to other markers often used in grapevine, like nuclear microsatellites (nuclear simple sequence repeats SSR), or chloroplast microsatellites (cpSSR) (Bourquin et al., 1993; Sefc et al., 2000; Arroyo-García et al., 2006).

Chloroplasts are maternally inherited in grapevine, and the analysis of polymorphisms in its genome permits to follow the maternal lineage of any vine. The analyses of chloroplast microsatellites in a large sample of wild and cultivated grapevines along the Mediterranean area allowed to identify four major chlorotypes (A, B, C, and D) with a differential geographic distribution among the wild *Vitis* populations analyzed (Arroyo-García et al., 2006). Chlorotypes B, C, and D were detected in Near and Middle Eastern populations, while mostly A chlorotype was found in Western Mediterranean populations, including Iberian populations. Interestingly, over 75% of Iberian (Spanish and Portuguese) varieties contain the A chlorotype, (Arroyo-García et al., 2006; Cunha et al., 2009; Castro et al., 2013), coincident with the chlorotype detected in their natural *sylvestris* populations. In addition, varieties bearing chlorotypes B, C, and D, found more frequently in Eastern wine varieties (B and D) and table grape varieties (C) (Arroyo-García et al., 2006; Laucou et al., 2018) were also found in Portuguese varieties (Cunha et al., 2009; Cunha et al., 2010; Castro et al., 2013; Cunha et al., 2015) supporting their multiple origins, likely including introductions from the Near East and from the Maghreb. Secondary domestication events have been proposed for grapevine along the Mediterranean basin based on differences in chlorotype frequencies (Grassi et al., 2003; Arroyo-García et al., 2006) or on the increased expected heterozygosity values detected further from the putative center of origin of the species (De Lorenzis et al., 2019).

Several genetic studies based on molecular markers have been carried out with Portuguese grapevines, proving to be powerful tools for cultivar identification, and resulting in the discovering of many cases of synonyms, homonyms and misnames. The existence of genetic relationships within cultivated and wild plants was surveyed, as well as pedigrees between autochthonous and foreign cultivars (Lopes et al., 1999; Lopes et al., 2006; Almandanim et al., 2007; Cunha et al., 2007; Cunha et al., 2009; Cunha et al., 2010; Veloso et al., 2010; Castro et al., 2011; Castro et al., 2013; Cunha et al., 2015; Cunha et al., 2016; Moita Maçanita et al., 2018). In a previous study, we genotyped 288 Portuguese grapevine accessions using a set of 48 SNP markers to detect synonyms, homonyms, and mistakes within the national germplasm collection (Cunha et al., 2016). Here we have increased the information on those accessions by genotyping them up to 261 SNP markers to carry out deeper genetic analyses aiming to: *i*) evaluate the genetic diversity and genetic structure of Portuguese germplasm; *ii)* identify first-degree genetic relationships using the SNP database of the Instituto de Ciencias de la Vid y del Vino (ICVV), which includes many varieties and wild plants; and *iii)* combine this information with chloroplast genotype information to help integrating historical data.

## Materials and Methods

### Plant Material

**Figure 1:**
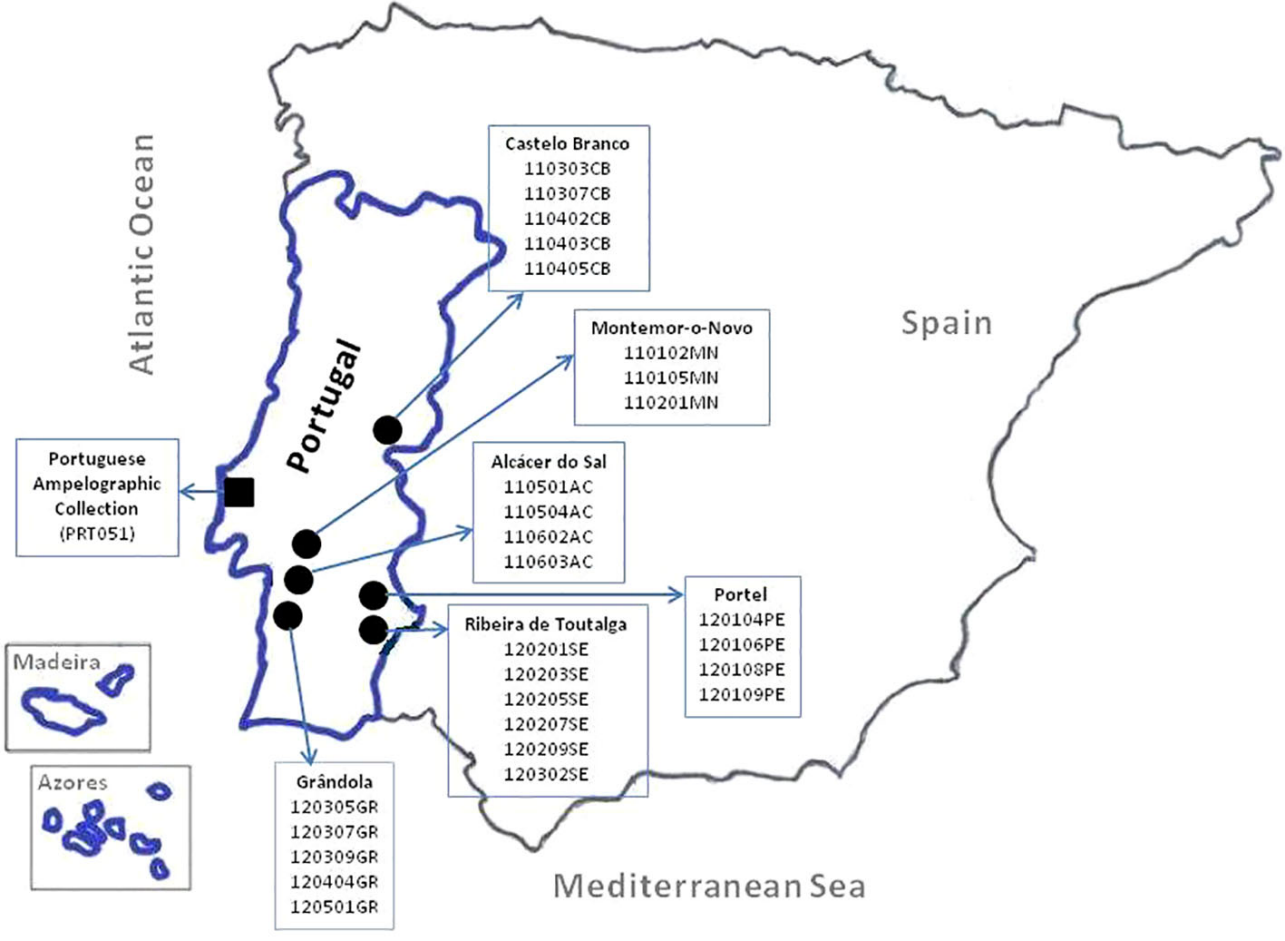
Map of Portugal with the locations of National Ampelographic Collection (◼) and wild vine populations *in situ* (●).

The Portuguese plant material used in this work was collected in the Portuguese National Ampelographic Collection (PRT051), which is the reference collection for varieties allowed in Portugal for wine production (MAMAOT, 2012). It is hosted by the Instituto Nacional de Investigação Agrária e Veterinária (INIAV) within the Portuguese Ministry of Agriculture, Forestry, and Rural Development. This collection was established in 1988 and it is located at Quinta da Almoinha, Dois Portos, Torres Vedras, Portugal [39°02′34.03″N, −9°10′57.41″W]. All the collection is grafted onto SO4 (Selection Oppenheim 4) rootstock and the training system is bilateral cordon (Royat). Two hundred and thirty-one accessions of *V. vinifera* L. ssp. *vinifera* (legally authorized to produce wine in Portugal) and 27 of *V. vinifera* L. ssp. *sylvestris* (C.C. Gmel.) Hegi [collected from six populations (Cunha et al., 2016) and maintained in the collection] were used (**Supplementary Table 1**). These samples were previously characterized using a set of 48 SNPs (Cunha et al., 2016) as well as with agronomical, morphological, and microsatellite markers (six and nine microsatellites) recommended by OIV (Veloso et al., 2010; Eiras-Dias et al., 2011; Eiras-Dias et al., 2013). The locations of the Portuguese National Ampelographic Collection and of the wild vine populations are shown in **Figure 1**. The other material used is the SNP database from the ICVV that includes genotypes from several different sources, mainly from the ICVV grapevine collection in La Grajera (ESP217, Logroño) and the Vitis Germplasm Bank (VGB) from the Instituto Madrileño de Investigación y Desarrollo Rural, Agrario y Alimentario (IMIDRA) in El Encín (ESP080, Alcalá de Henares). In addition, it also contains genotypes from several origins and collections: Algeria, Argentina, Australia, Belgium, Chile, France, Iran, Italy, Montenegro, Morocco, Portugal, Romania, Spain, and Tunisia.

### DNA Isolation

DNA was isolated from young leaves frozen at −80°C according to Thomas et al. (1993), with minor modifications. The quality and concentration of the DNA were determined by electrophoresis in agarose (0.8%) gels stained with ethidium bromide and visualized on a UV transilluminator. Concentration was calculated by comparing with known DNA concentrations (50, 100, and 200 ng/μl) of λDNA HindIII Fragments, 0.1 μg/μl, Invitrogen, Carlsbad, CA USA). NanoDrop 2000 C UV-Vis spectrophotometer (Thermo Scientific, Waltham, MA, USA) was used to check the quality and final concentration of each DNA sample.

### Single Nucleotide Polymorphism Analyses

DNA from grapevines was genotyped with 261 nuclear SNPs obtained by Lijavetzky et al. (2007) and Cabezas et al. (2011) (**Supplementary Table 2**). SNP genotyping was performed using SNPlex (Applied Biosystems, Waltham, MA USA) or Veracode (Illumina, San Diego, CA, USA) technologies as described in Zinelabidine et al. (2012; 2015). Genotyping services were provided in Spain by the National Genotyping Center^1^.

### Genetic Statistical Analysis

GeneAlEx 6.5 (Peakall and Smouse, 2012) was used to calculate the following genetic parameters: observed heterozygosity, expected heterozygosity, Shannon's information index, and hierarchical F-statistics. Calculations were performed on wild and cultivated groups separately using only polymorphic SNP markers in each group. The mean values of each parameter were analyzed using t-Student test (GraphPad Prism version 7.0, San Diego, CA, USA) to verify the statistical significance of the mean differences between the two groups. A *p*-value of <0.05 was considered statistically significant. Rarefaction on measures of allelic richness was performed to address the unbalanced sample size of wild and cultivated sets, using the program HP-RARE 1.0 (Kalinowski, 2005). GeneAlEx 6.5 was also used to test for deviation from the Hardy-Weinberg equilibrium (HWE) across all loci for each population.

Pairwise Euclidean distance was calculated for every pair of accessions using the genetic distance to assess the relationship among the wild and cultivated grapevine accessions, using the program GenAlEx 6.5.

MEGA 7 software, version 7.0.26 (Kumar et al., 2016), was used to generate a distance tree by the neighbor-joining (N-J) hierarchical clustering method (Saitou and Nei, 1987) based on the Pairwise Euclidean distance generated from the genetic distance obtained in GeneAlEx 6.5 software. Principal Coordinate analysis (PCoA) was performed on individual multilocus genotypes, with covariance standardized, using the same program.

Structure 2.3.4 (Pritchard et al., 2000) using the admixture model was employed to infer the number of genetic populations (K) existing in the sample and to assign individuals to their likely population of origin, with no prior information. A series of 10 independent runs was performed for each value of K between 1 and 15. An initial burn-in of 20,000 steps was used to minimize the effect of the starting configuration, followed by 100,000 Markov chain Monte Carlo steps, as recommended by Falush et al. (2007) and Ghaffari et al. (2014).

### Pedigree Analysis

The software CERVUS 3.0 (Kalinowski et al., 2007), that uses a likelihood-based method for computation, was used to identify first-order kinship relationships: trios (mother-father-offspring) and duos (parent-offspring pairs) among the studied (wild and cultivated) accessions. Pedigree analysis was done using as candidate parents a total of 1,921 profiles of different origins, including those obtained here, present in the ICVV-SNP database. Natural logarithm of the overall likelihood ratio—logarithm-of-odds (LOD) score—was calculated for each trio and duo identified. Chlorotypes previously determined for the Portuguese genotypes (Cunha et al., 2009; Castro et al., 2013) were used whenever possible to determine the maternal progenitor in the trio (Arroyo-García et al., 2006).

## Results

### Single Nucleotide Polymorphism Performance and Genetic Diversity

A total of 258 accessions (27 wild vines and 231 grapevine varieties) from the Portuguese Ampelographic Collection bearing non-redundant genotypes when analyzed with 48 SNPs (Cunha et al., 2016) were genotyped with up to 261 SNP markers. Thirty of these SNP markers were discarded: 28 because data were missing in 66% of the samples and two because genotyping failed in all samples. The results of the remaining 231 SNP markers were used for subsequent analyses (**Supplementary Table 1**).

The analyses of the genetic diversity of the Portuguese wild vines and grapevine varieties are presented in **Table 1**. Overall the genetic diversity parameters showed higher values in the cultivated group than in the wild set. In all cases these differences were statistically significant (*P* < 0.05) (**Table 1**). The estimated fixation index F (also called inbreeding coefficient F_IS_) in the wild genotypes showed a positive value of 0.109 ± 0.017, indicating a heterozygote deficiency in the wild group. In grapevine varieties, a mean negative value (−0.030 ± 0.008) of index F_IS_ was found, showing higher heterozygosity than in the wild samples. The analysis of allelic richness and private allelic richness using rarefaction methods, which take into account the differences in sample sizes, also showed a larger diversity in the cultivated set (**Table 1**).

**Table 1:** Summary of genetic diversity parameters estimated for *Vitis vinifera sylvestris* (wild vines) and *vinifera* (grapevine varieties) from Portugal.

Population	N		AR†	PAR†	Na	Ne	I	Ho	He	F
Wild vines	27	Mean	1.29	0.29	1.91	1.443	0.426	0,241	0.277	0.109
		SE	0.011	0.011	0.023	0.025	0.014	0.011	0.011	0.017
Grapevine varieties	231	Mean	1.35	0.34	2.000	1.593	0.521	0.361	0.348	-0.030
		SE	0.009	0.01	0.000	0.020	0.011	0.010	0.009	0.008
t test *P* value			0.0251*	<0.0001*	<0.0001*	0.0119*	0.0039*	<0.0001*	0.0082*	<0.0001*

N, sample size; AR†, allelic richness; PAR†, private allelic richness; Na, number of different alleles; Ne, number of effective alleles; I, Shannon's information index; Ho, observed heterozygosity; He, expected heterozygosity; F, fixation index; SE, standard error. Statistical significance according to t-Student test (*significant difference, p < 0.05). †Calculated using rarefaction methods.

Furthermore, the F_ST_ statistic of overall SNP loci was used to analyze the genetic differentiation among wild and cultivated subspecies and was estimated to be 0.158 when the total of the 258 accessions were used for the calculation.

Deviation from the Hardy-Weinberg proportion (*P* < 0.05) was observed for 54 (23.38%) markers in the cultivated group and for 32 (13.85%) markers in the wild set.

### Genetic Structure in Portuguese Wild and Cultivated Grapevines

**Figure 2:**
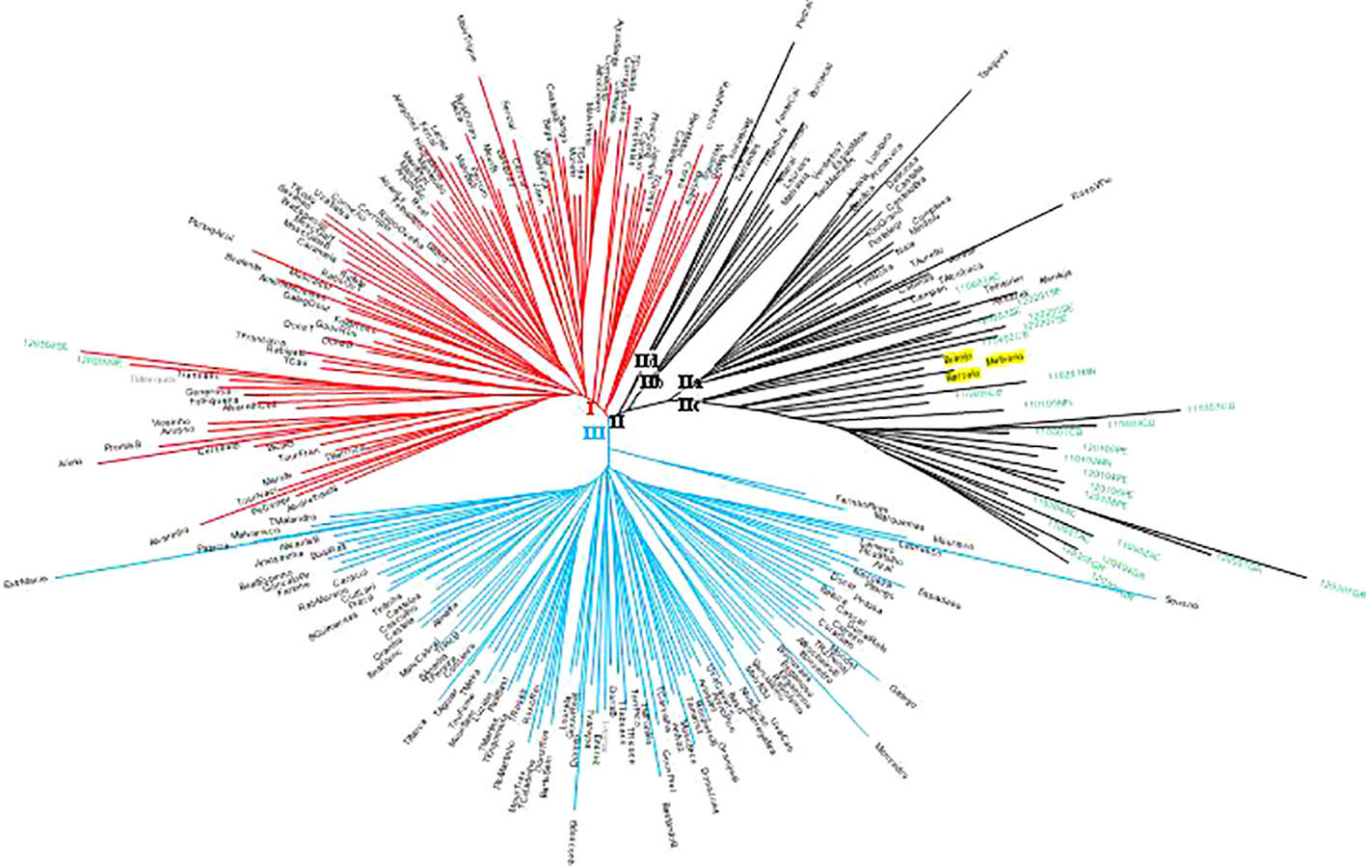
Neighbor-joining radiation tree showing genetic distance among Portuguese *Vitis vinifera* wild vines and varieties genotypes, based on 231 single nucleotide polymorphism (SNP) loci. Cluster I, solid lines in red color; cluster II, solid lines in black color; cluster III, solid lines in blue color; wild vine codes in green font; variety names in black font; cultivated grapevines with the wild group are highlighted in a yellow background.

A neighbor-joining (N-J) distance tree was constructed to investigate the genetic relationship among the 258 non-redundant genotypes from the 231 SNP matrix data (**Figure 2**). The hierarchical clustering of 258 unique genotypes produced three clusters (I, II, III). Cluster I mostly includes ancient Portuguese varieties and ancient Western and Central European varieties introduced and cultivated in Portugal time ago (e.g., Bastardo/Trousseau Noir, #12668; Branco Valente/Heunisch Weiss, #5374). Exceptions in cluster I are two wild vines, a male plant (120209SE) and a female plant (120302SE), from the population of Ribeira de Toutalga (Guadiana river basin, **Figure 1**). Cluster III groups mainly Portuguese varieties, including most of Marufo descendants and Bastardo descendants (see below). Apart from Fernão Pires, the most spread white variety in Portugal, most of the varieties included in this cluster are minority varieties. Cluster II includes four subgroups: subcluster IIa, with varieties obtained by the breeder Leão Ferreira de Almeida in the middle of 20th century; subcluster IIb, mostly with varieties from the Vinhos Verdes wine area (Northwestern Portugal); subcluster IIc, with almost all wild vines, and subcluster IId, with ancient varieties. Subcluster IIa also includes one wild vine genotype (110603AC) from the population of Alcácer do Sal (Sado river basin). In the subcluster IIc three varieties Barcelo, #980; Branjo, #17661 and Melhorio, #17225 cluster together with most wild vines. Two of these varieties, Barcelo and Melhorio, form duos, and thus are closely related, with Amaral, #818 (**Supplementary Table 3**). Amaral had already been mentioned in 1532 in the North of Portugal (Fernandes, 1532), while Barcelo was cited in the 18th century by Lacerda Lobo (1790).

**Figure 3:**
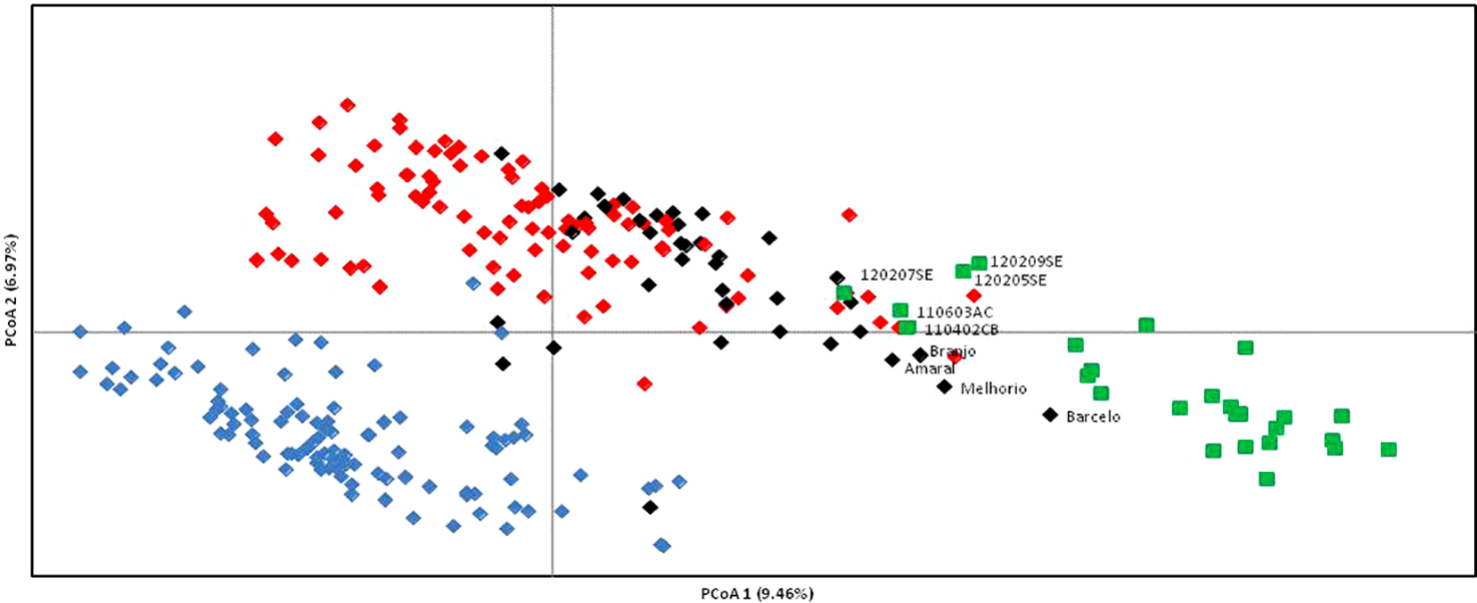
Plot of Portuguese *Vitis vinifera*, wild vines (green squares), and grapevine varieties (black, blue, and red diamonds) from a Principal Coordinate Analysis based on 231 single nucleotide polymorphism (SNP) markers and *via* covariance matrix with data standardization. Only wild and cultivated grapevines in intermediate positions are labeled. To facilitate the comparison with **Figure 2** the varieties are colored according to their position in **Figure 2** (red, black and blue for cluster I, II, and III respectively).

A non-hierarchical PCoA based on the square distances' matrix was also used to analyze the relationships between wild and cultivated grapevines as revealed by SNP markers (**Figure 3**). The first two principal axes explain only 16.43% of the total variation (9.46 and 6.97%, respectively). PCoA provides a similar result to the N-J distance analysis, separating the grapevine varieties in two groups (A, C) and a third group (B) with wild vines and cultivated genotypes, although this last group is not as clearly separated. Accessions from wild vine populations 120207SE, 110603AC, and 110402CB are in the edge of one of the clusters of grapevine varieties, while Barcelo is the closest variety to the wild vines. Amaral, Branjo, and Melhorio are also in the vicinity of the wild plants.

**Figure 4:**
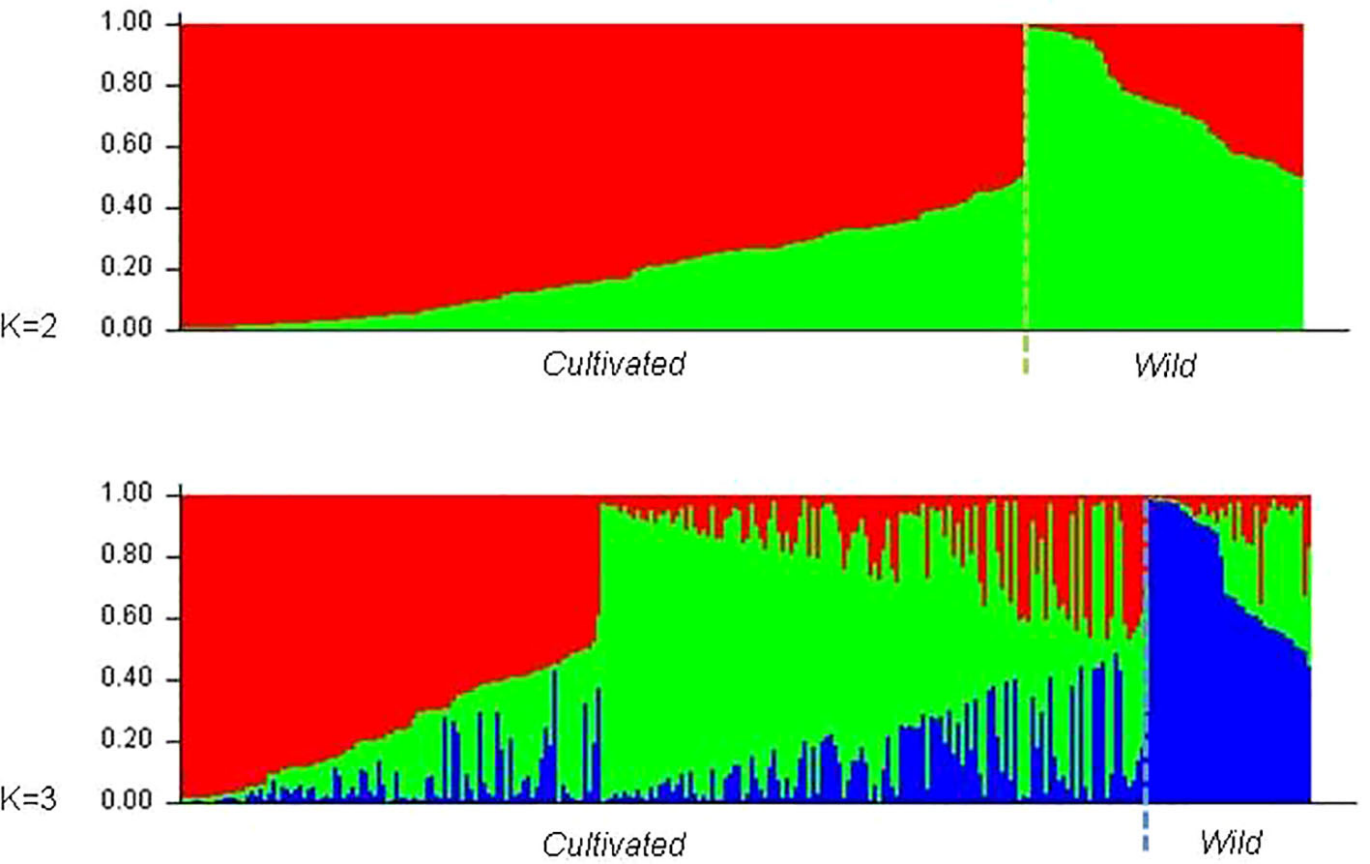
Barplot of the genetic population admixture in wild vines and cultivated varieties, as inferred by structure at K = 2 and K = 3. Each individual is represented by a single vertical bar broken into K color segments, with lengths proportional to the estimated probability of membership in each inferred cluster.

The existence of different number of genetic groups was also explored using Structure, from K = 1 to K = 15. Results for K = 2 and K = 3 were selected following Evanno et al. (2005) criterion and are displayed in **Figure 4**. In both cases 25 of the 27 wild plants were assigned to one of the subpopulations obtained (Pop2 and Pop3 respectively, **Supplementary Table 4**). The two accessions of wild vines that were not assigned to those groups were 120207SE and 110603AC. In the first scenario (K = 2), 39 out of the 231 grapevine varieties (17%) were assigned to Pop2, the “wild” subpopulation, including Amaral, Barcelo, Alvarinho (all with membership coefficients above 0.75), Branjo and Melhorio, and a few non-local varieties (Espadeiro Mole/Manseng Noir, #7340; Mondet/Durif, #3738; Santareno/Etraire de la Dui, #3993; Sevilhão/Corbeau, #2826 and Uva Salsa/Chasselas Cioutat, #2476). In the second scenario (K = 3), the subpopulation including most of the wild plants (Pop3) also included 12 cultivated genotypes, 11 of them Portuguese varieties such as the mentioned Amaral, Barcelo, and Alvarinho. The two other inferred subpopulations in K = 3 contain 54 and 41.6% of the grapevine varieties. Even though they do not match exactly, the groups assigned by structure (K = 3) mostly concord with the previously shown analyses of the N-J tree (**Figure 2**), and PCoA (**Figure 3**).

### Pedigrees of *Vitis vinifera* L. Within Portugal Germplasm and Local Origin of Varieties

**Figure 5:**
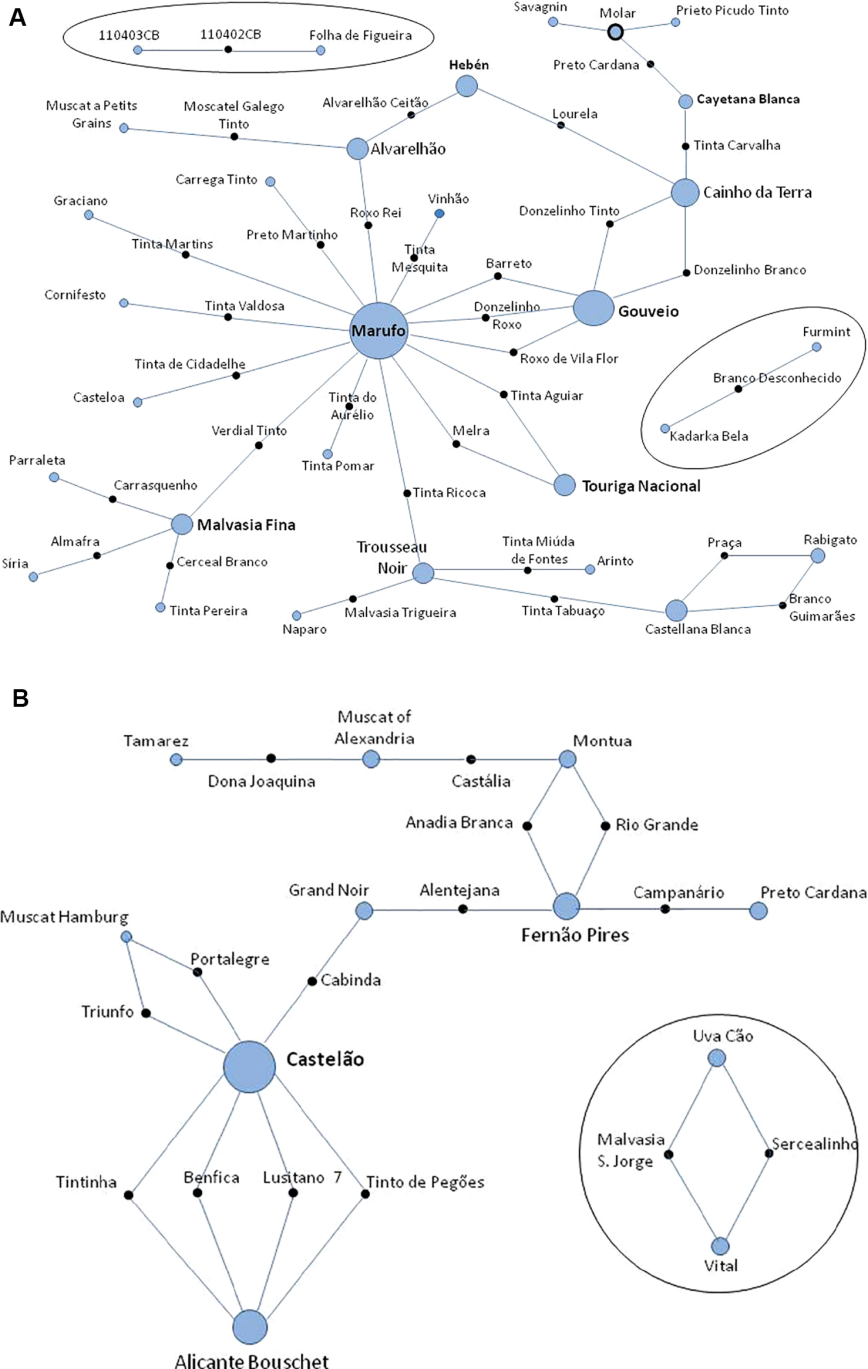
Networks of the proposed trios (parents and offspring) found in a parentage analysis of Portuguese set of *Vitis vinifera* germplasm using 261 single nucleotide polymorphism (SNP) markers. **(A)** Thirty-two previously unknown genetic relationships of varieties and/or wild plants; **(B)** confirmation of 15 pedigrees of new varieties reported by Almeida breeder in 1950. Solid black circles represent the offspring; blue circles represent the progenitors; circle diameter is proportional to the number of relationships where is involved; blue circles surrounded by a solid black line represent a variety that is an offspring and parent in the figure. The tree is not drawn to scale.

The data from 231 different varieties and 27 wild unique genotypes was added to the ICVV-SNP database, which was raised to 1,921 non-redundant genotypes, originated from the Near-East to Western European countries. A search for the possible first-order kinship relationships for these 258 non-redundant Portuguese genotypes was performed using all the 261 SNPs. One hundred and two trios (both parents and offspring) were identified (**Supplementary Table 5**). Thirty-two new trios are reported here for the first time, and all are supported by high LOD scores, that ranged from 52.70 to 80.70 (**Figure 5A** and **Supplementary Table 5**). Fifteen trios from the crosses made by the breeder Almeida (Ghira et al., 1982) were verified and all are supported by high LOD scores, that ranged from 61.5 to 85.3. Four of these bred varieties were the result of crosses between Castelão, #2324 and Alicante Bouschet, #304 (**Figure 5B** and **Supplementary Table 5**). Up to 55 trios previously described by other authors were confirmed (trios and references in **Supplementary Table 5**). Several hundred compatible duos (parent-offspring relationships) were also identified but, given the existence of large and close families among the studied accessions, it is difficult to conclude which ones correspond to real parent-offspring relationships. Only those considered more relevant are reported and discussed below (**Supplementary Table 3**).

For 65% of the Portuguese genotypes none compatible trio were found, i.e., their two parents could not be identified. A number of varieties were found to have played an important role in the genetic network of the studied grapevine varieties. Together with Alfrocheiro, #277; Mourisco Branco/Hebén, #5335 and Sarigo/Cayetana Blanca, #5648, previously described (Zinelabidine et al., 2012; Zinelabidine et al., 2015; Cunha et al., 2015), Marufo, #8086, a female grapevine variety bearing chlorotype (chl) D, was found to be a parent (mother) in 18 trios (14 identified for the first time in this study) (**Figure 5A**, **Supplementary Table 5**). Gouveio, #12953 (chl A), an old variety from Douro wine region, is involved as a parent in five new trios. Bastardo/Trousseau Noir, #12668 (chl A), Cainho da Terra (chl D), and Malvasia Fina, #715 (chl A) are parents in four new trios (**Figure 5A** and **Supplementary Table 5**). Alvarelhão, #1650 (chl A) and Castellana Blanca, #26280 (chl A) participated in three new trios. Touriga Nacional, #12594 (chl A); Sarigo (chl A); Mourisco Branco (chl A); Rabigato, #9857 (chl A); Uva Cão, #12812 (chl A) and Vital, #13122 (chl A) are each involved in two previously unknown trios (**Figure 5** and **Supplementary Table 5**).

A trio was found indicating gene flow from cultivated to wild population. The wild accession 110402CB, is a descendant of a cross between Folha de Figueira, a hermaphrodite variety from Castelo Branco region and 110403CB, a female wild plant (**Figure 5A**). The reliability of the trio is high as the LOD score was 69.80. The flowers of the accession 110402CB show a fully developed gynoecium and straight stamens shorter than the gynoecium (IPGRI, descriptor 6.2.1, note 4: female with straight stamens) in opposition to totally female plants that have reflexed stamens (IPGRI, descriptor 6.2.1, note 5). Folha de Figueira (*V*IVC #14142), is a white variety, which was first described by Lacerda Lobo in 1790. These wild plants were found in the margins of Ponsul river, a Northern tributary of the Tagus river, flowing close to the border with Spain in the Castelo Branco district.

Three consistent duos (0–1 mismatches, LOD > 25) involving wild vines were also identified. One duo is made up by the plants 110504AC and 110602AC from the Alcácer do Sal population, both males with different chloroplast haplotypes (A and B, respectively). The two other duos also involved cultivated varieties: one in the same Pônsul population between a female plant 110405AC and Frankenthal/Schiava Grossa, #10823 and the other in the Alcácer population between 110603AC and Castelão, #2324. Schiava Grossa is a spread variety from Italy, with 163 synonyms in the *V*IVC database (Maul et al., 2019). The oldest known reference in Portugal dates back from 1887, in the collection of Quinta da Viscondessa in Torres Vedras (Anonymous, 1887). Castelão is one of the most cultivated varieties in the south of Portugal (Cunha et al., 2015). Castelão wines are already mentioned in legal documents from the 14th century (Amaral, 1994).

## Discussion

The uneven sample sizes of the cultivated and wild sets of genotypes studied makes difficult the comparison of the genetic diversity parameters between them. Nevertheless, the results obtained, including those considering the sample sizes, indicate what other previous works have pointed out: the existence of a reduced diversity in the *sylvestris* subspecies (Marrano et al., 2017; Marrano et al., 2018). This unexpected situation (*sylvestris* subspecies is obligate outcrossing while most cultivars are self-pollinating) is caused by the small sizes commonly found in wild populations, due to their isolation by natural barriers, human actions, and the severe bottleneck that began in the 19th century with the pathogens introduced from North America (powdery mildew, downy mildew, and phylloxera) that has converted in relict the surviving populations.

### Portuguese Grapevine Varieties Are Structured in Three Major Genetic Groups

Taken together, all the genetic analyses performed on the Portuguese grapevine germplasm based on SNP genotypes indicate the existence of three major clusters or genetic groups. Genetic group I and III contain mostly cultivated genotypes while genetic group II includes most wild vines as well as cultivated genotypes from the region of Vinhos Verdes. Genetic group I includes cluster I defined in the N-J distance analysis (**Figure 2**), corresponding to PCoA group A (**Figure 3**) and cluster 2 in the structure analysis at K3 (green color in **Figure 4**). It grouped the ancient Portuguese varieties, with unknown parentage, together with foreign varieties contributing to the Portuguese germplasm either from Western and Central Europe, like Bastardo (BastT/Trousseau Noir); or Northern Africa, like Ferral (Ferral/Ahmeur bon Ahmeur); and varieties from the Near East, like Moscatel Galego Branco (MoscGaleB/Muscat a Petits Grains Blancs). Genetic group III includes cluster III of the N-J distance analysis (**Figure 2**), corresponding to PCoA group C (**Figure 3**) and cluster 1 in the structure analysis at K3 (red color in **Figure 4**). It grouped Portuguese varietal families identified in this or in previous works (Lopes et al., 1999; Cunha et al., 2015). Finally, genetic group II includes cluster II of the N-J distance analysis (**Figure 2**), corresponding to PCoA group B (**Figure 3**) and includes almost all wild plants, varieties obtained by Leão Ferreira de Almeida and ancient varieties from the Vinhos Verdes wine region in the Northwest of Portugal, like Amaral, Barcelo, Branjo e Melhorio. When considering the structure analysis at K =3 the ancient varieties from Vinho Verde region are still assigned to the genetic group containing most wild plants (**Supplementary Table 4**). Although the correspondences are not complete, the genetic group I would be similar to the S-5.3 (wine—West and Central Europe), while the genetic group III would be closer to S-5.1 (wine and table—Iberian Peninsula and Maghreb) as defined by Bacilieri et al. (2013).

A deeper analysis of these genetic groups points out the existence of close parentage relationships within specific varietal families. In fact, about a quarter of the studied genotypes were found to have kinship with Portuguese or Iberian varieties, as part of duos and/or trios. These include 37 descendants of two female varieties: Marufo (chl D) and Mourisco Branco (Hebén) (chl A), with 19 and 18 offspring respectively (**Figure 5A** this work; Lacombe et al., 2013; Zinelabidine et al., 2015). The N-J tree, the PCoA and the population analyses grouped these plants with the *vinifera* genotypes. The female condition obliges them to cross-pollinate, contributing to generate higher genetic diversity and heterozygosity in their offspring and a concomitantly increased plant vigor. This fact probably favored them having a larger number of descendants becoming new cultivated varieties. Mourisco Branco (Hebén) is the female parent of Sarigo (Cayetana Blanca) which has several descendants with its own mother (Zinelabidine et al., 2012; Zinelabidine et al., 2015; Cunha et al., 2015).

A restricted number of the studied grapevine varieties (near 10%) have one or both parents probably coming from outside the Iberian Peninsula. One of these varieties is the unexpected case of Branco Desconhecido (**Figure 5A**), an offspring of Furmint and Kadarka Bela, two Hungarian grapevine varieties. A different case is that of Savagnin, a very old and disseminated variety (105 synonyms in *V*IVC) that has been cultivated at least during the last 900 years (Ramos-Madrigal et al., 2019). Savagnin has had a strong impact in the genetic composition of Western and Central European varieties producing many descendants (Lacombe et al., 2013). In addition, two offspring varieties of Savagnin, Alfrocheiro, and Bastardo, were found to contribute several offspring to the Portuguese germplasm (Cunha et al., 2015; Ramos-Madrigal et al., 2019; **Figure 5A** and **Supplementary Table 5**). The dissemination of Savagnin and Bastardo, the later already referred by Fernandes (1532) in the Douro region, could be explained by the extended medieval network formed by the Benedictines and Cistercians monasteries all over Europe. Cistercian monks had important and influential monasteries with vineyards in the Douro area (São João de Tarouca and Santa Maria de Salzedas) and its Portuguese headquarter was Santa Maria de Alcobaça (UNESCO world heritage monument), 110 km North of Lisbon. Remarkably, most of the Portuguese varieties that derive from Bastardo and Savagnin are found in the Douro and Lisbon regions. Direct germination of seeds and recollection of naturally growing plants was a documented practice in Portugal (Alarte, 1712; Fonseca, 1791) and it is likely in the origin of its present large grapevine genetic diversity. Variety names like Mourisco de Semente (*V*IVC #12471) and Barreto de Semente, synonym of the Barreto variety (*V*IVC #17655) further suggest the idea of plant multiplication through seed germination since “de Semente” means in Portuguese “from seed.”

Almost half of the varieties analyzed in this study could not be assigned to any trio or duo within the ICVV-SNP database. Since this database includes a large collection of Iberian (both Spanish and Portuguese) genotypes, we think that it is highly probable that their progenitors were not conserved either because they were lost during the phytopathological crisis of the 19th century or because they were minor varieties or individual plants lost along the evolution of viticulture.

### Evidences of Possible Introgression of *Sylvestris* Into Cultivated Germplasm and *Vice-Versa* in Portugal

It is very difficult to establish strong conclusions on the genetic relationships between cultivated and wild grapevine plants given the exiguous number of currently available wild populations. Still, several results from the described analyses suggest the existence of gene flow between wild populations and cultivated plants. In this way, grouping of varieties Barcelo, Branjo, Melhorio, and Amaral with *sylvestris* plants within cluster II (**Figure 2**), and/or in the PCoA analysis (**Figure 3**), and/or in the genetic groups containing most wild accessions for K=2 (Pop2) and for K=3 (Pop3) suggest a close genetic relationship among these cultivated varieties and wild plants. In fact, phenotypically, these four varieties share some trait similarity with wild plants: small and very loose bunches with many visible pedicels, small berries with blue black skin color, high acidity, and small orbicular leaves with one or three lobes. In addition, Barcelo and Amaral form a duo (LOD 45.76), as well as Melhorio and Amaral (LOD 25.18) (**Supplementary Table 3**). Interestingly, Amaral is clustered with wild plants in all studies and has several descendants among Portuguese varieties, as shown here (**Supplementary Tables 3** and **5**) and in previous works (Díaz-Losada et al., 2011; Lacombe et al., 2013). The association of these varieties used in the Vinhos Verdes region with the wild vines points out to the existence of introgression from wild plants into currently cultivated varieties in Portugal. Interestingly, wines from this region have low alcohol content, high acidity, and are naturally sparkling (http://www.vinhoverde.pt/en/demarcated-region, features characteristic of must or wine obtained from wild grapes: low sugar content, low pH, and high tartaric acid content (Arroyo-Garcia et al., 2006; Cunha et al., 2007). These results are in agreement with Riaz et al. (2018) whose findings indicated a considerable amount of gene flow between the two subspecies, which limited their differentiation, and the contribution of Western European wild populations to the development of Western European wine grapes.

The close genetic relationship between Vinhos Verdes varieties and wild plants is especially remarkable because the wild populations are not from that region, where wild plants have not been found. Anthropogenic pressure is probable the main cause of the current absence of wild vines populations in the Vinhos Verdes region, as it is the case in many other regions. At present, only in the south of Portugal it was possible to find wild populations surviving in riparian woods along small streams (Cunha et al., 2007).

On the other side, the degree of introgression from ancient and current cultivated varieties into the actual wild populations cannot be estimated but it is exemplified by the existence of wild plants grouped within clusters of cultivated ones (**Figure 2**), and by the relatively low values of membership coefficient to the wild group of the plants of the population from Ribera de Toutalga (**Supplementary Table 4**). The human use of *V. vinifera* ssp. *sylvestris* continued uninterrupted until the late 20th century to produce vinegar and folk medicines, as well as for other uses not related to grapes or wine (Ocete et al., 1999). This continuous use probably favored obtaining new varieties through selection of interesting individuals produced from seeds, in some cases perhaps from spontaneous hybrids between cultivated and *sylvestris* plants, such as those found in this work, which could inherit the hermaphrodite allele (50%) favoring their selection.

In our case a dubious, intermediate, flower phenotype was found in the accession 110402CB, which is a descendant of a cross between Folha de Figueira and 110403CB, a female wild plant from the Ponsul river population (**Figure 5A**). Apart from this full pedigree, two putative parent-offspring relationships (**Supplementary Table 3**) were also identified involving cultivated and wild plants: one in the same Ponsul population between a female plant 110405CB and Schiava Grossa and the other in the Alcácer do Sal population between female plant 110603AC (same type of flower as plant 110402CB) and Castelão. These three grapevines, sampled as “wild” (110402CB, 110405CB, and 110603AC) are grouped in cluster II (subcluster a) (**Figure 2**) or in intermediate positions close to *vinifera* genotypes (**Figure 3**) and represent clear cases of introgression into the *sylvestris* subspecies. There are other two grapevines collected in the wild populations (120209SE a male plant and 120302SE, female plant with straight stamens) that grouped in the cluster I (**Figure 2**), or in intermediate positions (**Figure 3**) and for which no kinship relationships were found. They could correspond to feral forms with morphological characteristics of wild plants. Introgression of cultivated into wild plants threatens the large potential of *sylvestris* as a source of resilience factors in future breeding programs to deal with climate change and the increasing demand of a sustainable viticulture (Marrano et al., 2018).

Grapevine domestication is probably a long process taking place along thousands of years and within a wide geographical area. Some authors defend the existence of a single domestication event, with later wild introgression events for local adaptation (Zhou et al., 2019) while others take the view of a primary domestication event in Transcaucasia and later secondary events of domestication in the West (Grassi et al., 2003; Arroyo-García et al., 2006). These two views are not so far, because both subspecies are not separate compartments in nature and gene flow has always occurred between them (Di Vecchi-Staraz et al., 2009) and because, given the relevance of vegetative multiplication in grapevine, the number of generations separating cultivated varieties from wild plants is likely very small (This et al., 2006; Arroyo-García et al., 2006). This is shown by the small genetic and phenotypic differences between wild and cultivated plants observed in some cases like those found in this work. The wild vines analyzed here belong to populations still present in the Tagus, Guadiana and Alcácer do Sal river basins, where palynological data of Paúl dos Patudos, Alpiarça (upstream the Tagus valley), showed a 33% increase of *Vitis* pollen in a stratum of the 7th century BCE compared to earlier strata. This led the authors, when related it to other evidences of anthropogenic changes in the palynological structure of the deposits, to consider it to be due to grapevine cultivation. Records predating 6050 BCE are certainly from subspecies *sylvestris* (Leeuwaarden and Janssen, 1985).

Moreover, seed remains found in northern part of Portugal have a Stummer index between 0.65 and 0.75 that could be assigned to a mix of both *vinifera* and *sylvestris* subspecies, as well as, seeds remains found in Iron Age site in Castro Marim (Algarve) (Ramil Rego and Aira Rodríguez, 1993; Queiroz and Mateus, 1994; Aira Rodríguez and Ramil Rego, 1995; Sanches, 1997; Queiroz and Mateus, 2007). The existence of a large particular gene pool of Portugal varieties that bear chlorotype A means that the seeds originating these cultivars (or her mothers, or her grandmothers) were produced in *sylvestris* plants, and thus human beings collected and multiplied plants grown from seeds of *sylvestris* plants, which had germinated and raised in the wild, and so they “domesticated” (select, collect, and cultivate) such plants. All these findings point out to the possible genetic contribution of wild to local cultivated grapevines, as it was shown in France for most archeological samples analyzed by Ramos-Madrigal et al. (2019) and it was indicated for wine Western European cultivars by Riaz et al. (2018).

## Conclusions

The identification of kin relationships allowed the identification of major contributors to the present Portuguese germplasm, in particular the contribution of female genotypes. An important contribution for the Portuguese germplasm consists in the descendants of Savagnin (chl D) (Alfrocheiro, Bastardo, and Molar) probably introduced by Cistercian monks. Despite some foreign contributions, exemplified by the descendance of Marufo (chl D), about half of the Portuguese grapevine varieties analyzed in this study hold the A chlorotype, typical of the Iberian Peninsula and have no known kinship, while a quarter of the genotypes have some sort of kinship with Portuguese or Iberian varieties. The high number of varieties resulting from different parents seems to reflect a wide traditional use of seeds for vine propagation in Portugal.

The analysis of the SNP genotypes of the Portuguese cultivated and wild germplasm allowed to detect the existence of gene flow between both subspecies probably since historical times and until near present. This is corroborated by the disclosure of wild-cultivated kinship relations, especially in the South of Portugal where wild populations are still present in riparian woods. This subspecies has suffered a severe reduction in its genetic diversity due to the nineteenth century phytopathological crisis and to the anthropogenic pressure, hindering the study of its domestication. The introgression in the wild plants observed in this work illustrates the magnitude of the problem and reinforce the need to protect, study, and use the existing wild germplasm, which may be a source of useful characteristics to incorporate in the Portuguese germplasm.

The genetic relationships, ampelographic similarities, and must and wine characteristics share between the Vinhos Verdes region varieties and the wild vines, the specific genetic pool with chlorotype A existing in Portugal and different archaeological findings support the possible existence of secondary domestication events in Portugal, or, at least, of an introgression process of wild into cultivated grapevines.

## Data Availability Statement

All datasets generated and analyzed for this study are included in the article/**Supplementary Material**.

## Author Contributions

JC, JI, and JE-D conceived and designed the experiments. JC and JI performed the experiments. JC, JI, and MT-S analyzed the data. JC, JI, MT-S, JB, PF, JM-Z, and JE-D wrote the manuscript. JE-D, JC, and JB established *vinifera* and *sylvestris* collections and collected plant material. All authors contributed to editing the final version of the manuscript.

## Conflict of Interest

The authors declare that the research was conducted in the absence of any commercial or financial relationships that could be construed as a potential conflict of interest.

## Footnotes

1 http://www.cegen.org
